# A critical assessment of clustering algorithms to improve cell clustering and identification in single-cell transcriptome study

**DOI:** 10.1093/bib/bbad497

**Published:** 2024-01-02

**Authors:** Xiao Liang, Lijie Cao, Hao Chen, Lidan Wang, Yangyun Wang, Lijuan Fu, Xiaqin Tan, Enxiang Chen, Yubin Ding, Jing Tang

**Affiliations:** Department of Obstetrics and Gynecology, Women and Children’s Hospital of Chongqing Medical University, Chongqing 401147, China; School of Basic Medicine, Chongqing Medical University, Chongqing 400016, China; School of Basic Medicine, Chongqing Medical University, Chongqing 400016, China; School of Basic Medicine, Chongqing Medical University, Chongqing 400016, China; School of Basic Medicine, Chongqing Medical University, Chongqing 400016, China; School of Basic Medicine, Chongqing Medical University, Chongqing 400016, China; Joint International Research Laboratory of Reproduction and Development of the Ministry of Education of China, School of Public Health, Chongqing Medical University, Chongqing 400016, China; Department of Pharmacology, Academician Workstation, Changsha Medical University, Changsha 410219, China; The First Affiliated Hospital of Chongqing Medical University, Chongqing 400016, China; School of Basic Medicine, Chongqing Medical University, Chongqing 400016, China; Joint International Research Laboratory of Reproduction and Development of the Ministry of Education of China, School of Public Health, Chongqing Medical University, Chongqing 400016, China; Department of Obstetrics and Gynecology, Women and Children’s Hospital of Chongqing Medical University, Chongqing 401147, China; Joint International Research Laboratory of Reproduction and Development of the Ministry of Education of China, School of Public Health, Chongqing Medical University, Chongqing 400016, China; Department of Obstetrics and Gynecology, Women and Children’s Hospital of Chongqing Medical University, Chongqing 401147, China; School of Basic Medicine, Chongqing Medical University, Chongqing 400016, China

**Keywords:** single-cell RNA sequencing, clustering algorithms, deep learning, performance evaluation, cell identification

## Abstract

Cell clustering is typically the initial step in single-cell RNA sequencing (scRNA-seq) analyses. The performance of clustering considerably impacts the validity and reproducibility of cell identification. A variety of clustering algorithms have been developed for scRNA-seq data. These algorithms generate cell label sets that assign each cell to a cluster. However, different algorithms usually yield different label sets, which can introduce variations in cell-type identification based on the generated label sets. Currently, the performance of these algorithms has not been systematically evaluated in single-cell transcriptome studies. Herein, we performed a critical assessment of seven state-of-the-art clustering algorithms including four deep learning-based clustering algorithms and commonly used methods Seurat, Cosine-based Tanimoto similarity-refined graph for community detection using Leiden’s algorithm (CosTaL) and Single-cell consensus clustering (SC3). We used diverse evaluation indices based on 10 different scRNA-seq benchmarks to systematically evaluate their clustering performance. Our results show that CosTaL, Seurat, Deep Embedding for Single-cell Clustering (DESC) and SC3 consistently outperformed Single-Cell Clustering Assessment Framework and scDeepCluster based on nine effectiveness scores. Notably, CosTaL and DESC demonstrated superior performance in clustering specific cell types. The performance of the single-cell Variational Inference tools varied across different datasets, suggesting its sensitivity to certain dataset characteristics. Notably, DESC exhibited promising results for cell subtype identification and capturing cellular heterogeneity. In addition, SC3 requires more memory and exhibits slower computation speed compared to other algorithms for the same dataset. In sum, this study provides useful guidance for selecting appropriate clustering methods in scRNA-seq data analysis.

## INTRODUCTION

Single-cell RNA sequencing (scRNA-seq) is a popular and powerful technology that enables the profiling of the whole transcriptome of a large number of individual cells [1, 2]. Compared with bulk RNA-seq, scRNA-seq has the following characteristics: high sensitivity [3], unbiasedness [4, 5], dynamic monitoring [6] and enabling to detect individual differences [7]. ScRNA-seq has brought important biological insights and discoveries, which can be applied for facilitating the understanding of development of different tissues and organs [8], revealing spatial and functional heterogeneity in tumor microenvironment [9, 10], investigating host–pathogen interactions under maximum containment [11, 12], and providing a promising solution for studying intercellular communication and signal transduction in cardiovascular diseases [13].

In the data processing protocols of scRNA-seq experiments, cell-type identification is a vital step for subsequent analysis [14, 15]. A typical strategy for cell-type identification is cluster-based annotation, indicating that all cells were first clustering by graph-based clustering and then used differentially expressed marker genes at the level of pre-computed clusters to annotate the clusters [14]. However, the process of cell-type identification using graph-based clustering may encounter challenges in accurately separating different types of cells, potentially resulting in the inability to distinguish certain cell types. This may be attributed to the following: batch effects cannot be effectively removed [16]; scRNA-seq data are sparse (due to low RNA capture rate) [17]; clustering algorithm resolution depends on subjective judgment [18] and the increasing number of cells which may reach thousands to millions [19].

To address the issue of unreliable clustering results, caused by the aforementioned problems, a variety of state-of-the-art clustering methods have been developed in single-cell transcriptome study, which included four deep learning-based clustering algorithms such as Deep Embedding for Single-cell Clustering (DESC) [19], single-cell Variational Inference tools (scVI) [20], scDeepCluster [21] and Single-Cell Clustering Assessment Framework (SCCAF) [22]. In addition to their application in investigating cell states and phenotypes in scRNA-seq data [23, 24], deep learning methods have gained popularity and been extensively applied in other bioinformatics fields, including predicting chromatin loops [25], chromatin interactions [26] and promoters [27]. Moreover, there have been recent developments of other clustering algorithms specifically designed for scRNA-seq data analysis, such as Single-cell consensus clustering (SC3) [28], PARC [29], Cosine-based Tanimoto similarity-refined graph for community detection using Leiden’s algorithm (CosTaL) [30] and SCMcluster [31]. Since clustering algorithms are sensitive to sequencing platforms [32] and dropouts [33], the results of different clustering algorithms can also vary greatly. Even if the same algorithm is used to process different data, as the points representing cells in the scRNA-seq data become more similar when represented in high-dimensional space, the performance of clustering algorithms will also be affected by data from different dimensions [34, 35]. Thus, it is crucial to evaluate the performance of these diverse clustering algorithms to improve cell clustering and identification in single-cell transcriptome study.

Currently, several existing clustering algorithms have been evaluated and compared in different studies. For instance, Duò *et al*. [36] performed a systematic assessment of 14 clustering algorithms and found that SC3 and Seurat exhibited the most favorable cluster results. Stassen *et al*. developed a new algorithm called PARC [29] and compared its performance against other state-of-the-art clustering algorithms, including PhenoGraph, FlowSOM and Flock, and demonstrating that PARC consistently outperformed them. Li *et al*. [30] introduced a novel algorithm called CosTaL, which was shown to outperform PhenoGraph, Scanpy and PARC. Wu *et al*. [31] developed the SCMcluster algorithm, which utilized cellular marker genes and demonstrated that superior performance compared to other clustering methods. However, there is still a lack of comprehensive comparison and evaluation between these well-performing clustering algorithms and deep learning-based clustering algorithms in current studies.

Herein, using 10 scRNA-seq datasets as benchmarks (involved into three types of biological backgrounds), we conducted a systematic performance evaluation of various clustering algorithms including Seurat, DESC, scVI, scDeepCluster, SCCAF, CosTaL and SC3 from multiple perspectives. Based on the comparative results in terms of clustering number, diverse evaluating indices measuring clustering effectiveness and computation efficiency, we demonstrate that CosTaL, Seurat, DESC and SC3 consistently perform well, particularly on clustering effectiveness measured by nine evaluation metrics across different types of benchmarks. scVI exhibits good clustering performance in some datasets while performing poorly in others. The performance of the scVI varied across different datasets in terms of these evaluating indices. While scDeepCluster and SCCAF exhibit poor performance compared to other algorithms, their clustering performance have substantial variations across different benchmarks. Moreover, DESC exhibited promising results for cell subtype identification and capturing cellular heterogeneity. When applied to the same scRNA-seq dataset, SC3 requires more memory and exhibits slower computation speed compared to other algorithms. This study provides a useful guidance for selecting appropriate clustering methods for scRNA-seq data analysis.

## MATERIALS AND METHODS

### Benchmark datasets collection for single-cell clustering algorithm comparison

We evaluate clustering algorithms on 10 scRNA-seq benchmark datasets selected from the GEO and ArrayExpress databases (Table 1). Based on the culture system and cell type of the data, we further classify the data into three categories: for Type 1: different culture systems for the same cell type (GSE196091 and GSE189120); for Type 2: the same culture system for different cell types (GSE180914, GSE159183, GSE89497, GSE171381, E-MTAB-6701); and for Type 3: different cell types and different culture systems (GSE194209, GSE139850, GSE171993). The details of each benchmark dataset are provided in the Supplementary Data.

**Table 1:** Ten scRNA-seq datasets were utilized to systematically analyze and compare seven single-cell clustering algorithms

**Dataset ID**	**Sample source**	**Cell label**	**Case type**	**Expression unit**	**Cell (gene)**
**GSE196091**	*Homo sapiens*	Label known by experiment	Type 1: Different culture systems for the same cell type	Reads count	31 730 (20 028)
**GSE189120**	*Homo sapiens*	Label known by experiment	Type 1: Different culture systems for the same cell type	Reads count	25 511 (20 838)
**GSE180914**	*Pleurodeles waltl*	Label generated by scRNA-seq	Type 2: The same culture system for different cell types	Reads count	681 (16 545)
**GSE159183**	*Mus musculus*	Label known by experiment	Type 2: The same culture system for different cell types	Reads count	738 (24 711)
**GSE89497**	*Homo sapiens*	Label known by experiment	Type 2: The same culture system for different cell types	TPM	1567 (21 465)
**GSE171381**	*Homo sapiens*	Label generated by scRNA-seq	Type 2: The same culture system for different cell types	Reads count	83 378 (25 127)
**E-MTAB-6701**	*Homo sapiens*	Label generated by scRNA-seq	Type 2: The same culture system for different cell types	Reads count	64 734 (26 212)
**GSE194209**	*Mus musculus*	Label known by experiment	Type 3: Different cell types and different culture systems	Reads count	20 393 (17 454)
**GSE139850**	*Homo sapiens*	Label known by experiment	Type 3: Different cell types and different culture systems	Reads count	14 874 (22 401)
**GSE171993**	*Mus musculus*	Label known by experiment	Type 3: Different cell types and different culture systems	Reads count	52 834 (24 043)

The datasets GSE196091 and GSE189120 were classified as Type 1 cases, while GSE180914, GSE159183, GSE89497, GSE171381 and E-MTAB-6701 were classified as Type 2 cases. Lastly, GSE194209, GSE139850 and GSE171993 were classified as Type 3 cases.

### Clustering algorithms utilized for performance evaluation in this study

In this work, seven cell clustering algorithms were analyzed (Supplementary Table S1), including a typically traditional Seurat method, four state-of-the-art deep learning-based clustering algorithms such as DESC, scVI, single-cell Model-based Deep Embedded Clustering (scDeepCluster), SCCAF, CosTaL and SC3. The instructions on these clustering algorithms are provided in the Supplementary Methods.

### Diverse evaluating indices used for determining performance of clustering algorithm

In this study, different types of metrics were used for assessing the performance of clustering algorithm. These evaluation metrics included t-SNE plot & clustering number, and nine effectiveness scores and computation speed & memory usage. These diverse evaluation metrics included Adjusted Rand Index (ARI), Homogeneity Score (H Score), Kullback–Leibler (KL) Divergence, Normalized Mutual Information (NMI), Adjusted Mutual Information (AMI), FlowCAPI F1 score (FF1), Hungarian algorithm-based F1 score (HF1), Fowlkes–Mallows Index (FMI) and V-measure. The characterizations of these diverse evaluation metrics are demonstrated in Supplementary Table S2. First, a graphical representation of performance, such as dimension reduction and data visualization using t-SNE [37], was utilized. Herein, we evaluated the ability of clustering algorithms in identifying cell subtype and characterizing cellular heterogeneity using a single metric CN (clustering number), which were obtained by the algorithm for a given cell type. Second, the true labels for the cells were defined based on the well-known cellular information provided by the original publications in this study. These true labels represent the known cell types or experimental conditions associated with each cell. By using these true labels as a reference, the performance of the clustering algorithms can be evaluated and compared. The predictive labels for the cells were defined using a majority voting scheme [38]. Briefly, clustering algorithm usually partitions data into a certain number of clusters, patterns in the same cluster should be similar to each other, while patterns in different clusters should not [39]. We obtained all the clusters generated by each algorithm’s clustering result. For each cluster, the number of cells with the same true label is counted, and the true label with the highest count is selected as the predictive label for that cluster. This approach ensures the predictive label represents the main characteristic of the cells within that cluster, thereby enabling a more accurate evaluation of the clustering algorithm’s performance.

## RESULTS AND DISCUSSIONS

### Study overview

**Figure 1:**
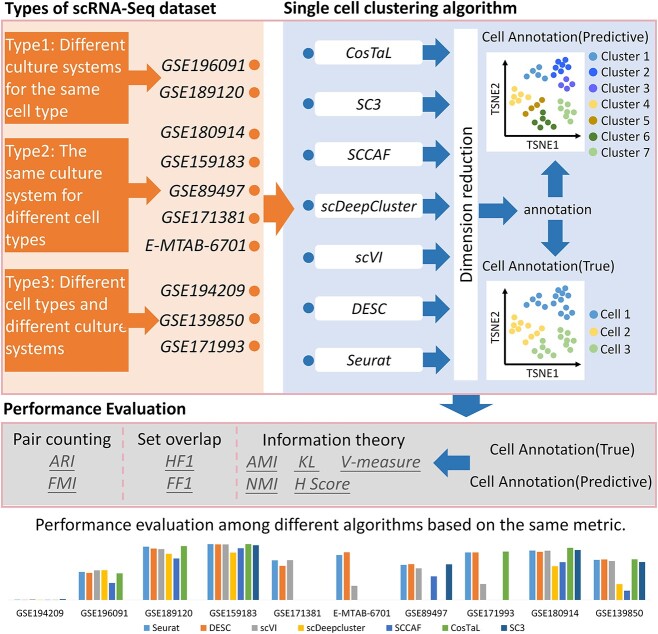
The overview of this study framework. The framework is divided into three major parts: types of scRNA-seq dataset, single-cell clustering algorithm and performance evaluation. The true labels of cells are cell identifiers from corresponding previous publications, while the predictive labels represent cell identifiers generated by the clustering algorithm. The performances of seven clustering algorithms were assessed utilizing nine metrics.

Diverse clustering algorithms were evaluated using 10 scRNA-seq datasets. Seurat was able to identify distinct clusters, but the clusters were found to be highly sensitive to the choice of resolution parameter, where higher values tend to result in a larger number of clusters [40, 41]. Deep learning clustering algorithms have emerged as promising approaches to address challenges such as batch effects in scRNA-seq data, and were shown to be effective algorithm for cell clustering and identification [23, 24]. In addition, there are other clustering algorithms specifically designed for single-cell clustering analysis, including SC3 and CosTaL. We adopted nine metrics (three categories) to evaluate performance of algorithms. An overview of the framework for comparing diverse algorithms are shown in Figure 1.

### Performance evaluation of clustering algorithms using t-SNE plot and clustering number

**Figure 2:**
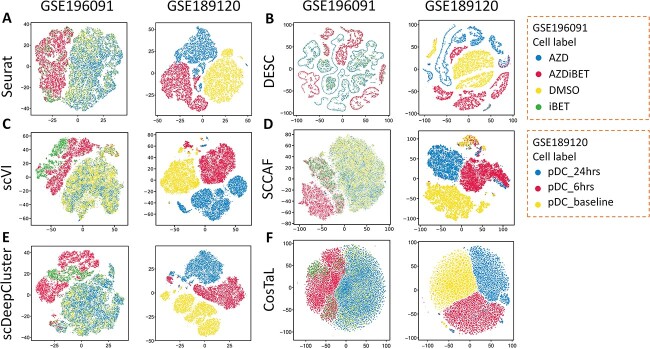
Performance comparison of clustering results for Type 1 data across six algorithms. (**A**–**F**) The t-SNE plots show the visualization of clustering results for Type 1 data using Seurat (**A**), DESC (**B**), scVI (**C**), SCCAF (**D**), scDeepCluster (**E**) and CosTaL (**F**).

**Figure 3:**
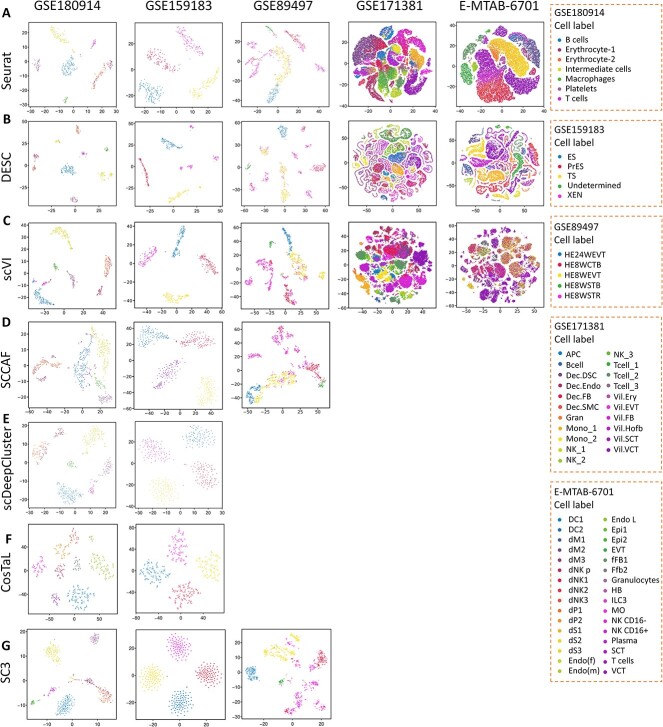
Performance comparison of clustering results for Type 2 data across seven algorithms. (**A**–**G**) The t-SNE plots show the visualization of clustering results for Type 2 data using Seurat (**A**), DESC (**B**), scVI (**C**), SCCAF (**D**), scDeepCluster (**E**), CosTaL (**F**) and SC3 (**G**).

**Figure 4:**
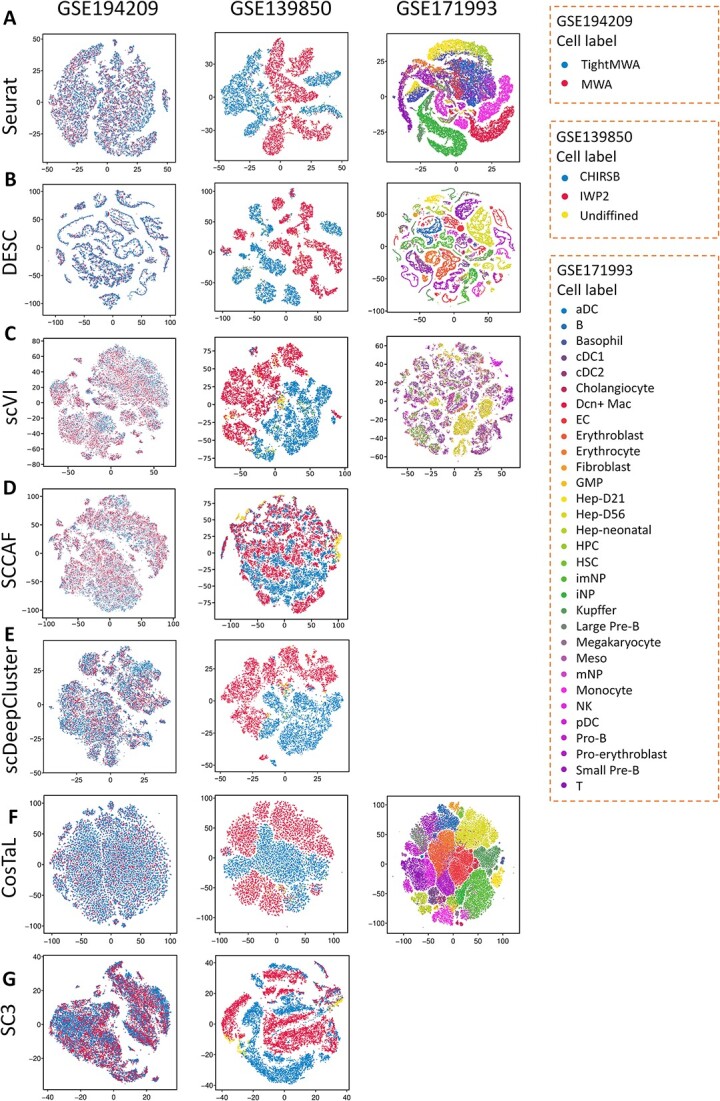
Performance comparison of clustering results for Type 3 data across seven algorithms. (**A**–**G**) The t-SNE plots show the visualization of clustering results for Type 3 data using Seurat (**A**), DESC (**B**), scVI (**C**), SCCAF (**D**), scDeepCluster (**E**), CosTaL (**F**) and SC3 (**G**).

In the context of identifying cell subtypes and cell heterogeneity, the number of clusters generated by clustering algorithms can serve as an indicator of the algorithm’s ability to accurately capture the underlying cellular heterogeneity [42]. A greater number of clusters suggests that the clustering algorithm is effective in distinguishing distinct subtypes within a given cell type [42]. For instance, in the case of placental cytotrophoblasts cells (CTBs), the generation of three subtypes by algorithm indicates that CTBs exhibit both high and varying proliferative capacities [43]. To compare clustering algorithms, we refer to clustering number (showing distinct and separated clusters) as an evaluation measure for evaluating the ability of algorithms in identifying cell subtype and heterogeneity. Figures 2, 3 and 4 show the t-SNE plots illustrating clustering results for all types of datasets using seven algorithms.

### Application to datasets of different culture systems for the same cell type (Type 1)

Figure 2 shows the visualization of clustering results for *Type 1* data across diverse algorithms. In GSE196091, all algorithms exhibit poor performance in clustering N6 cells under different treatments, particularly for N6 cells in AZD medium (blue) and N6 cells in DMSO medium (yellow). These results can be attributed to the fact that N6 cells under different treatments do not exhibit significant differences at the overall gene expression level. In GSE189120, the three true labels (pDC_24hrs, pDC_6hrs and pDC_baseline) were well separated in all algorithms. Notably, as shown in Figure 2(B), the DESC algorithm can cluster the data into multiple subgroups for the same cell type. For example, DESC exhibits more distinct as well as separated cell clusters for pDC_6hrs cells (red) compared to other algorithms. Although the Seurat generated most the numbers of clusters (Supplementary Figure S1), these clusters do not display obvious separation. This may be attributed to resolution parameter. The t-SNE plots of clusterings for *Type 1* can be seen in Supplementary Figure S1. Overall, for *Type 1* datasets, DESC results often yield more clustering subgroups showing obvious separation compared to other algorithms for the same cell type. These results show that DESC display good performance in identifying cell subtypes and cell heterogeneity. Due to the memory usage limitations, the SC3 do not obtain comparable cluster results.

### Application to datasets of the same culture system for different cell types (Type 2)

Figure 3 shows the visualization of clustering results for *Type 2* data using seven algorithms. As demonstrated in Figure 3, for all five datasets, all clustering algorithms obviously separated the distinct cell types. For example, in GSE180914, seven cell types including B cells, erythrocyte-1, erythrocyte-2, intermediate cells, macrophages, platelets and T cells display obvious separation. The clustering results of these seven algorithms in GSE159183 are consistent with the true label, which obviously divided these cells into ES, PrES, TS and XEN types. Notably, DESC (Figure 3B) exhibits superior performance in clustering HE8WEVT and HE24WEVT cells (for GSE89497), Dec_DSC and Dec_FB cells (for GSE171381), as well as NK CD16^+^ and dNK cells (for E-MTAB-6701). In contrast, Seurat and scVI fail to effectively cluster these cell types. Due to memory limitations, the SCCAF, scDeepCluster, CosTaL and SC3 algorithms do not obtain comparable cluster results for GSE171381 and E-MTAB-6701. In addition, it is important to note that the GSE89497 dataset only provides normalized data in transcripts per million (TPM) and does not include Read Count expression information in Table 1. Thus, we did not compare the performance of CosTaL and scDeepCluster for this dataset in this study. Moreover, as shown in Figure 3(B), the DESC algorithm can cluster the data into multiple subgroups for the same cell type. For example, DESC exhibits more distinct as well as separated cell clusters for erythrocyte-2 (GSE180914), XEN (GSE159183) and HE8WEVT (GSE89497) compared to other algorithms. The t-SNE plots of clusterings for *Type 2* can be seen in Supplementary Figure S2. In sum, these results indicate that DESC display good performance in identifying cell subtypes and cell heterogeneity.

### Application to datasets of different cell types and different culture systems (Type 3)

Figure 4 shows the visualization of clustering results for *Type 3* data using seven algorithms. As demonstrated in Figure 4, in the GSE194209 dataset, we observed that none of the algorithms were able to effectively separate the CD45^+^ cells (isolated from tumor tissue) between TightMWA and MWA treatments*.* In GSE139850, SCCAF performed poorly in distinguishing cells (isolated from embroid body) under IWP2 and CHIRSB treatments. In GSE171993, DESC and CosTaL performed well in distinguishing different cell types (isolated from different postnatal days), especially exhibiting the good performance in distinguished B cells and T cells (Figure 4B and F), whereas Seurat and scVI fail to separate these two cell types. Moreover, as shown in Figure 4(B), the DESC algorithm can cluster the data into multiple subgroups for the same cell type. For example, DESC exhibits more distinct as well as separated cell clusters for CHIRSB (GSE139850) and EC (GSE171993) compared to other algorithms. The t-SNE plots of clusterings for *Type 3* can be seen in Supplementary Figure S3. These results indicate that DESC displays good performance in identifying cell subtypes and heterogeneity. Due to the limitation of a large number of cells (for GSE171993), SCCAF, scDeepCluster and SC3 did not produce comparable clustering results.

Overall, these findings demonstrate that six algorithms performed well in clustering different cell types across most benchmark datasets, with the exception of SCCAF. DESC and CosTaL consistently exhibited strong performance across diverse datasets, particularly for specific cell types. DESC could be a well-suited method for cell subtype identification and cellular heterogeneity, as it effectively clusters cells of the same type in a distinct and separated manner. This factor contributing to DESC’s this performance may be the use of a biologically interpretable clustering assignment probability in DESC, which can reveal the discrete and pseudo-time structure of cells [19].

### Performance evaluation of clustering algorithms using effectiveness scores

To evaluate the effectiveness of the clustering algorithms, we performed a quantitative evaluation using nine metrics, including ARI, H Score, KL Divergence, NMI, AMI, FF1, HF1, FMI and V-measure. These metrics are widely used to assess the classification ability of clustering algorithms and are acknowledged to provide a comprehensive performance evaluation. A higher value of ARI, H Score, NMI, AMI, FF1, HF1, FMI and V-measure indicates better performance of the algorithm. A lower value of KL represents better performance of the algorithm. The characteristics of all metrics used in this study are demonstrated in Supplementary Table S2. Based on the categories adopted by Li *et al.*, we classified the nine metrics into three distinct categories: Pair counting, Set overlap and Information theory [30].

#### Performance of metrics (pair counting)

**Figure 5:**
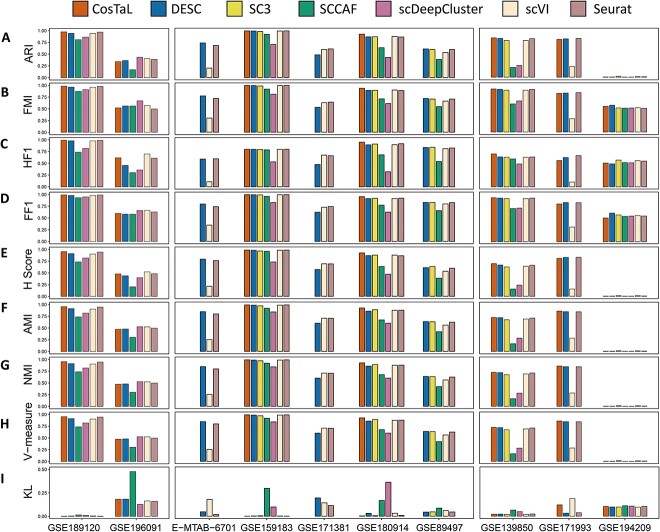
Performance comparison of different clustering algorithms based on nine evaluation metrics. (**A**) ARI: Type 1 (left), Type 2 (middle), Type 3 (right). (**B**) FMI: Type 1 (left), Type 2 (middle), Type 3 (right). (**C**) HF1: Type 1 (left), Type 2 (middle), Type 3 (right). (**D**) FF1: Type 1 (left), Type 2 (middle), Type 3 (right). (**E**) H Score: Type 1 (left), Type 2 (middle), Type 3 (right). (**F**) AMI: Type 1 (left), Type 2 (middle), Type 3 (right). (**G**) NMI: Type 1 (left), Type 2 (middle), Type 3 (right). (**H**) V-measure: Type 1 (left), Type 2 (middle), Type 3 (right). (**I**) KL: Type 1 (left), Type 2 (middle), Type 3 (right).

Figure 5(A) and (B) illustrates the comparison of ARI and FMI among seven algorithms. The results represent that CosTaL, DESC, Seurat and SC3 algorithms have consistently superior performance in terms of ARI and FMI for most datasets, while SCCAF and scDeepCluster have consistently poor performance for these datasets. However, the scVI algorithm showed inconsistent performance across different datasets. For example, the ARI and FMI scores of scVI were lowest in E-MTAB-6701 and GSE171993, while it exhibited the higher level in GSE180914, GSE171381, GSE139850 and GSE159183. In addition, the ARI and FMI scores of SCCAF were higher in GSE159183 and GSE180914 than that of scDeepCluster, but in GSE189120, GSE196091 and GSE139850 was lower. Interestingly, in GSE196091, the performance of scDeepCluster was even better than that of CosTaL and DESC. All ARI and FMI scores are shown in Supplementary Tables S3–S4.

#### Performance of metrics (set overlap)

Figure 5(C) and (D) illustrate the comparison of HF1 and FF1 among seven algorithms. The higher HF1 and FF1 scores of CosTaL, Seurat, DESC and SC3 showed that they outperformed for most datasets. The HF1 and FF1 scores of SCCAF and scDeepCluster are consistently low for most datasets, which indicate that SCCAF and scDeepCluster exhibit poor performance across different datasets. scVI has a low HF1 and FF1 in E-MTAB-6701 and GSE171993, but high in the other datasets. These findings suggested that scDeepCluster and scVI exhibit inconsistent performance across different datasets. All HF1 and FF1 scores are shown in Supplementary Tables S5–S6.

#### Performance of metrics (information theory)

Figure 5(E)–(H) illustrates the comparison of H Score, NMI, AMI and V-measure among seven algorithms. Similar to metrics based on Pair counting and Set overlap, for H Score, NMI, AMI and V-measure, the higher scores of CosTaL, Seurat, DESC and SC3 showed that they outperformed for most datasets. The scores of SCCAF and scDeepCluster are consistently low for most cases, which indicate SCCAF and scDeepCluster exhibit poor performance across these datasets. scVI has low scores in E-MTAB-6701 and GSE171993, but high in the other datasets. These findings suggested that scDeepCluster and scVI exhibit inconsistent performance across different datasets. Moreover, Figure 5I shows a comparison of KL among seven algorithms. The lower KL scores of CosTaL, Seurat, DESC and SC3 showed that they outperformed across all datasets. The KL value of SCCAF and scDeepCluster is consistently high across all datasets, which indicates that SCCAF and scDeepCluster exhibit poor performance for most cases. scVI has a high KL value in E-MTAB-6701 and GSE171993, but low in the other datasets. These findings suggested that scVI exhibits inconsistent performance across different datasets. All H Score, NMI, AMI, V-measure and KL Divergence scores are shown in Supplementary Tables S7–S11.

Notably, in GSE194209 and GSE196091, all algorithms produced very low scores (ARI, H Score, NMI, AMI, FF1, HF1, FMI and V-measure) compared to other datasets, especially GSE194209. This may be attributed to the CD45^+^ cells isolated from tumor tissue between TightMWA and MWA treatments that do not exhibit obvious biological variation (GSE194209). N6 cells treated by between AZD and DMSO medium do not exhibit significant differences at the overall gene expression level (GSE196091). The performance of algorithms based on these scores is consistent with the t-SNE plots in Figures 2 and 4. This observation suggests that there may be a lack of distinct biological variations among cells under different conditions in these datasets. It is crucial to consider that the effectiveness scores may not fully capture the performance of clustering algorithms in such cases. These metrics primarily evaluate the agreement between the clustering results and the ground truth labels or known biological features. When the underlying biological variations are minimal or absent, it becomes challenging for any clustering algorithm to accurately identify meaningful clusters. Generally, all datasets that exhibit clear biological variation between distinct conditions are suitable for clustering analysis. On the other hand, datasets that do not show obvious biological variation may be not suitable for clustering analysis. Furthermore, for the different type datasets, CosTaL, Seurat, DESC and SC3 outperformed SCCAF and scDeepCluster. However, the performance of the scVI algorithm varied across different datasets. It showed good performance in some datasets while performing poorly in others. This variability suggests that the scVI algorithm may be more sensitive to certain dataset characteristics.

### Performance evaluation of clustering algorithms using computation speed and memory usage

To assess the performance of clustering algorithms in terms of computation speed and memory usage, we conducted a thorough analysis. As demonstrated in Supplementary Table S12, Seurat demonstrated the most superior computation speed compared to the other six clustering algorithms across all 10 benchmark datasets. DESC, SCCAF and CosTaL also exhibited good computation speed. However, as the volume of data increases, the computation speed of CosTaL became slower compared to the other two algorithms. On the other hand, scDeepCluster and scVI showed modest computation speed, while SC3 exhibited poor computation speed. For memory usage, SC3 required more memory for implementing the single-cell clustering for the same dataset. scDeepCluster and scVI required modest memory usage, while Seurat, DESC, SCCAF and CosTaL required minor memory usage for implementing the single-cell clustering. In addition, as expected, when the number of cells analyzed increases, the computation speed tends to slow down, and memory usage becomes larger. Furthermore, we released the source codes for seven single-cell clustering algorithms and nine effectiveness scores on the CAFCA website (http://rdblab.cn/cafca/). These codes are readily available for download, allowing users to access and utilize them on their local computers.

## CONCLUSIONS

We systematically compared the performance of clustering algorithms across 10 benchmark datasets using diverse evaluation metrics. Our results indicate that CosTaL, Seurat, DESC and SC3 consistently outperformed SCCAF and scDeepCluster based on nine effectiveness scores across most benchmark datasets. Notably, CosTaL and DESC demonstrated superior performance in clustering specific cell types. Furthermore, our evaluation metrics revealed variability in the performance of scVI, suggesting its sensitivity to certain dataset characteristics. On the other hand, DESC exhibited promising results for cell subtype identification and capturing cellular heterogeneity. This performance may be attributed to the use of a biologically interpretable clustering assignment probability in DESC, which reveals discrete and pseudo-time structures of cells. In addition, we compared the computation speed and memory usage of the algorithms. Our findings indicate that SC3 requires more memory and exhibits slower computation speed compared to other algorithms when applied to the same scRNA-seq dataset. Overall, our study provides valuable insights for researchers in selecting appropriate clustering algorithms based on their specific research needs. We highlight the strengths and limitations of each algorithm, considering factors such as performance, interpretability, computational efficiency, and suitability for different cell types and dataset characteristics.

**Key Points**

- A systematic comparison of different state-of-the-art single-cell clustering algorithms was conducted using 10 scRNA-seq benchmark datasets, which involved three types of case situations.
- To improve cell clustering and identification, the performance of these clustering algorithms was collectively assessed using a variety of well-established indices with distinct underlying theories.
- To capture the underlying biological complexity and heterogeneity of the cell population, clustering (showing distinct and separated clusters) generated for the same cell type was utilized.

## Supplementary Material

Tang-Supplementary_Materials_bbad497

## Data Availability

The source codes for seven single-cell clustering algorithms and the nine effectiveness scores are available at the CAFCA website (http://rdblab.cn/cafca/).

## Author Biographies

**Xiao Liang**, **Lijie Cao**, **Hao Chen** and **Lidan Wang** are master candidates in the School of Basic Medicine at Chongqing Medical University. They are working on single-cell analysis and web server construction.

**Lijuan Fu** is a professor in Chongqing Medical University. She is working on molecular mechanism, epidemiology and transformation of female reproductive-related diseases.

**Yangyun Wang** and **Xiaqin Tan** are undergraduates at Chongqing Medical University.

**Enxiang Chen** is a postdoctoral fellow in the School of Basic Medicine at Chongqing Medical University. He is interested in trophoblastic stem cells and organoids.

**Yubin Ding** is a professor in Women and Children’s Hospital of Chongqing Medical University. He is working on molecular mechanism, epidemiology and transformation of female reproductive related diseases.

**Jing Tang** is an associate professor in the School of Basic Medicine at Chongqing Medical University. She is interested in single-cell analysis and web server construction.
