# Supplementary material for: A critical assessment of clustering algorithms to improve cell clustering and identification in single-cell transcriptome study
Source: Brief Bioinform. 2024 Jan 2;25(1):bbad497. doi: 10.1093/bib/bbad497 (PMC10782910; doi:10.1093/bib/bbad497)
Supplement: Tang-Supplementary_Materials_bbad497 [file tang-supplementary_materials_bbad497.docx]

**Supplementary Materials for:**

**A Critical Assessment of Clustering Algorithms to Improve Cell Clustering and Identification in Single-cell Transcriptome Study**

Xiao LIANG**^1^**^,^**^2^**, Lijie CAO**^2^**, Hao CHEN**^2^**, Lidan WANG**^2^**, Yangyun WANG**^2^**, Lijuan FU**^3^**^,^**^4^**, Xiaqin TAN**^5^**, Enxiang CHEN**^2^**^,^**^3^**^,*^, Yubin DING**^1^**^,^**^3^**^,*^ and Jing TANG**^1^**^,^**^2^**^,*^

**^1^** Department of Obstetrics and Gynecology, Women and Children’s Hospital of Chongqing Medical University, Chongqing, 401147, China

**^2^** School of Basic Medicine, Chongqing Medical University, Chongqing, 400016, China

**^3^** Joint International Research Laboratory of Reproduction and Development of the Ministry of Education of China, School of Public Health, Chongqing Medical University, Chongqing 400016, China

**^4^** Department of Pharmacology, Academician Workstation, Changsha Medical University, Changsha, 410219, China

**^5^** The First Affiliated Hospital of Chongqing Medical University, Chongqing, 400016, China

**^*^** To whom correspondence should be addressed. Tel: +86 23 68485868; Fax: +86 23 68485868; Email: [tang_jing@cqmu.edu.cn](mailto:tang_jing@cqmu.edu.cn)

Correspondence may also be addressed to Enxiang CHEN, Email: [exchen@cqmu.edu.cn](mailto:exchen@cqmu.edu.cn); Yubin DING, Email: [dingyb@cqmu.edu.cn](mailto:dingyb@cqmu.edu.cn)

Running Title: Assessing scRNA-seq Data Clustering Algorithms

**Supplementary Data**

**Detailed information of scRNA-seq datasets**

For GSE196091 [1], the data was derived from N6 cells that were sequenced after being treated with different inhibitors, and were treated with DMSO, AZD, iBET and AZD plus iBET (AZDiBET) respectively. The annotation of culture system in this dataset was known during the experiment. There are 31730 cells in total, which belong to ***Type 1****: Different culture systems for the same cell type*. The resolution of algorithm clustering is set to 1.5 according to the number of cells.

For GSE189120 [2], the data was derived from plasmacytoid dendritic cells (pDCs) that were stimulated with influenza virus, and their temporal responses were examined at 0, 6, and 24 hours. The annotation of culture system in this dataset was known during the experiment. There are 25511 cells in total, which belong to ***Type 1****: Different culture systems for the same cell type*. The resolution of algorithm clustering is set to 1.5 according to the number of cells.

For GSE180914 [3], epicardium-derived cells of salamander were analyzed from 7 day post cryoinjury (dpci), including B cells, Erythrocyte, Intermediate, Macrophages, Platelets and T cells. The annotation of cell type in this dataset was generated by a typical scRNA-seq analysis workflow. There are 681 cells in total, which belong to ***Type 2***: *the same culture system for different cell types*. The resolution of algorithm clustering was set to 0.5 according to the number of cells.

For GSE159183 [4], the data was derived from mammalian blastocysts which consist of four different cell types: Ectoderm, Trophoblast, Primitive Endoderm Stem (PrES) cells, and Extraembryonic Endoderm Stem Cells (XEN). The annotation of cell type in this dataset was isolated and known during the experiment. There are 738 cells in total, which belong to ***Type 2***: *the same culture system for different cell types*. The resolution in algorithm clustering was set to 0.5 according to the number of cells.

For GSE89497 [5], The data was derived from human chorionic stroma cells (STR), CTB, STB and EVT that were isolated during the first three months and the second trimester of pregnancy. The annotation of cell type in this dataset was isolated and known during the experiment. There are 1567 cells in total, which belong to ***Type 2***: *the same culture system for different cell types*. The resolution of algorithm clustering was set to 0.5 according to the number of cells.

For GSE171381 [6], this dataset was derived from the decidua and placental villi of healthy control samples and SARS CoV-2 positive samples. A total of 21 cell types were identified in this dataset. The annotation of cell type in this dataset was generated by a typical scRNA-seq analysis workflow. There are 64734 cells in total, which belong to ***Type 2***: *the same culture system for different cell types*. The resolution of algorithm clustering was set to 2.0 according to the number of cells.

For E-MTAB-6701 [7], the data was derived from fetal cells obtained from maternal blood and decidua samples during the first trimester of pregnancy. The annotation of cell type in this dataset was generated by a typical scRNA-seq analysis workflow. There are 64734 cells in total, which belong to ***Type 2***: *the same culture system for different cell types*. The resolution of algorithm clustering is set to 2.0.

For GSE194209 [8], the data was derived from the MC38 tuner model under two different culture conditions, microwave ablation (MWA) and the combination of MWA and immunoreceptor tyrosine-based inhibitory motif domain (MWA+TIGIT). The annotation of culture system in this dataset was known during the experiment. There are 20393 cells in total, which belong to ***Type 3***: *different cell types and different culture systems*. The resolution of algorithm clustering is set to 1.5 according to the number of cells.

For GSE139850 [9], the data was obtained from the Embroid Bodies (EBs) on the third day, under two different culture conditions: WNTi (IWP2+ACTIVIN A) and WNTd (CHIR99021+SB43152). The annotation of culture system in this dataset was known during the experiment. There are 14874 cells in total, which belong to ***Type 3***: *different cell types and different culture systems*. The resolution of algorithm clustering was set to 1.5 according to the number of cells.

For GSE171993 [10], the hepatic cells (C57BL/6 livers) were isolated from the liver of mice on the 1st, 3rd, 7th, 21st and 56th days after birth. A total of 31 cell types were identified in this dataset. The annotation of cell type in this dataset was isolated and known during the experiment. There are 52834 cells in total, which belong to ***Type 3***: *different cell types and different culture systems*. The resolution of algorithm clustering was set to 2.0.

**Supplementary Methods**

**Detailed information of clustering algorithms**

**Seurat** [4], a traditional computational strategy that infers cellular localization by combining scRNA-seq data with in situ RNA model. Seurat can correctly localize rare subgroups, and accurately map spatially restricted and scattered groups. Seurat is particularly suitable for visualizing the cell localization in complex patterned tissues in diverse systems. The emergence of Seurat has provided a valuable research tool for fields such as developmental biology [11], epigenetics [12] and cancer mechanism research [13] that need to comprehend the origin and characteristics of different cell types in complex tissues. Seurat V4.3.0 was adopted in R 4.1.3, and default values were used for all parameters except for the clustering resolution.

**DESC**, as an unsupervised deep embedding algorithm, [14] utilizes iterative optimization of a clustering objective function to cluster scRNA-seq data. This algorithm provides a biologically interpretable clustering assignment probability, enabling the identification of discrete and pseudo-time structure within cells. One of the key benefits of DESC is its ability to balance clustering accuracy and stability, while utilizing low memory usage. DESC can effectively remove batch effects without requiring explicit batch processing information, and its compatibility with GPU acceleration. In addition, DESC can be applied to the biomedical field, especially in the analysis of human tissues, which is complex and cannot avoid the removal of batch effects [14]. DESC V2.1.1 was adopted in python 3.6.2, and default values were used for all parameters except for the clustering resolution.

**scVI** [15] is a fully probabilistic approach that enables joint representation and analysis of scRNA-seq data. This algorithm was developed to address the challenges posed by limited sensitivity and instability, batch effects, and transcription noise in single cell analysis. The scVI is based on the hierarchical Bayesian model [16, 17] of conditional distribution specified by the deep neural network. Even for very large datasets, it can be trained very effectively. However, the scVI cannot directly process non-Euclidean spatial data [18]. scVI performs relatively well in complex integrated tasks (such as tissue or organ maps) [19]. scVI V0.6.8 was adopted in python 3.7.11, and default values were used for all parameters except for the clustering resolution.

**scDeepCluster** [6] is a deep embedded clustering method based on single cell model, which combines zero-inflated negative binomial (ZINB) model with clustering loss in a principled way. Its principle is to map the read count matrix of scRNA-seq data to a low-dimensional potential representation using a nonlinear function, which is learned by an autoencoder based on the ZINB model. The clustering task is then performed in the potential space using the Kullback-Leibler (KL) divergence. To enhance the robustness of feature representation, scDeepCluster also incorporates denoising autoencoder technology[20] into the automatic encoder based on the ZINB model [21]. The developer thinks that it is more suitable for analyzing large scRNA-seq data [6]. scDeepCluster was adopted in python 3.6.3, and default values were used for all parameters.

**SCCAF** [22] is an iterative application of clustering based on machine learning and self-projection, so as to gradually merge clusters that can be defined by similar sets of feature genes. Its improved skip connection structure "skip connection based on cross-attention fusion" mechanism can effectively reduce the semantic difference between encoder features and decoder features, thereby improving feature fusion performance. It is worth mentioning that SCCAF solves the problem of traditional clustering algorithms, which tend to over-cluster larger subgroups when raise the resolution and fail to identify smaller subgroups when reduce the resolution. Therefore, in this clustering analysis, we select the most suitable round as the final predictive label during iterations, based on the number of true labels assigned to each data. SCCAF V2.1.1 was adopted in python 3.7.0, and default values were used for all parameters except for the clustering resolution.

SC3 is a user-friendly tool that achieves high accuracy and robustness in unsupervised clustering by combining multiple clustering solutions through a consensus approach[23]. SC3 utilizes a parallelization method, thereby simultaneously evaluating important subsets of the parameter space to obtain a set of clusters. The final result provided by SC3 is determined by performing complete-linkage hierarchical clustering of the consensus matrix into k groups[23]. SC3 clustering algorithm may face the problems of computational complexity and memory consumption when dealing with large-scale datasets[24]. SC3 V1.22.0 was adopted in R 4.1.0, the parameter “rand_seed” was set to 1, “ks” was set to the k value which estimated in preprocessing, “svm_max” was set to the cell number of datasets, the other parameters were set to default values.

CosTaL is a graph-based clustering method that transforms cells with high-dimensional features into a weighted k-nearest-neighbor (kNN) graph[25]. CosTaL does not need normalization, scaling or principal component analysis (PCA) transformation when clustering the scRNA-seq dataset, which significantly reduces the work involved in parameter selection and improves the overall clustering efficiency. CosTaL is known for its efficiency on large datasets, making it a highly scalable algorithm compared to other methods. This scalability is particularly advantageous for large-scale analyses[25]. CosTaL was adopted in python 3.11.4, the parameter “seed” was set to 1, “method” was set as “tani”, and “pp_method” was set as “scrnaseq”, the other parameters were set to default values.

**Supplementary Figure S1**. The t-SNE plots generated using six clustering algorithms for Type 1 data. Different colors represent clusters generated by clustering algorithms.


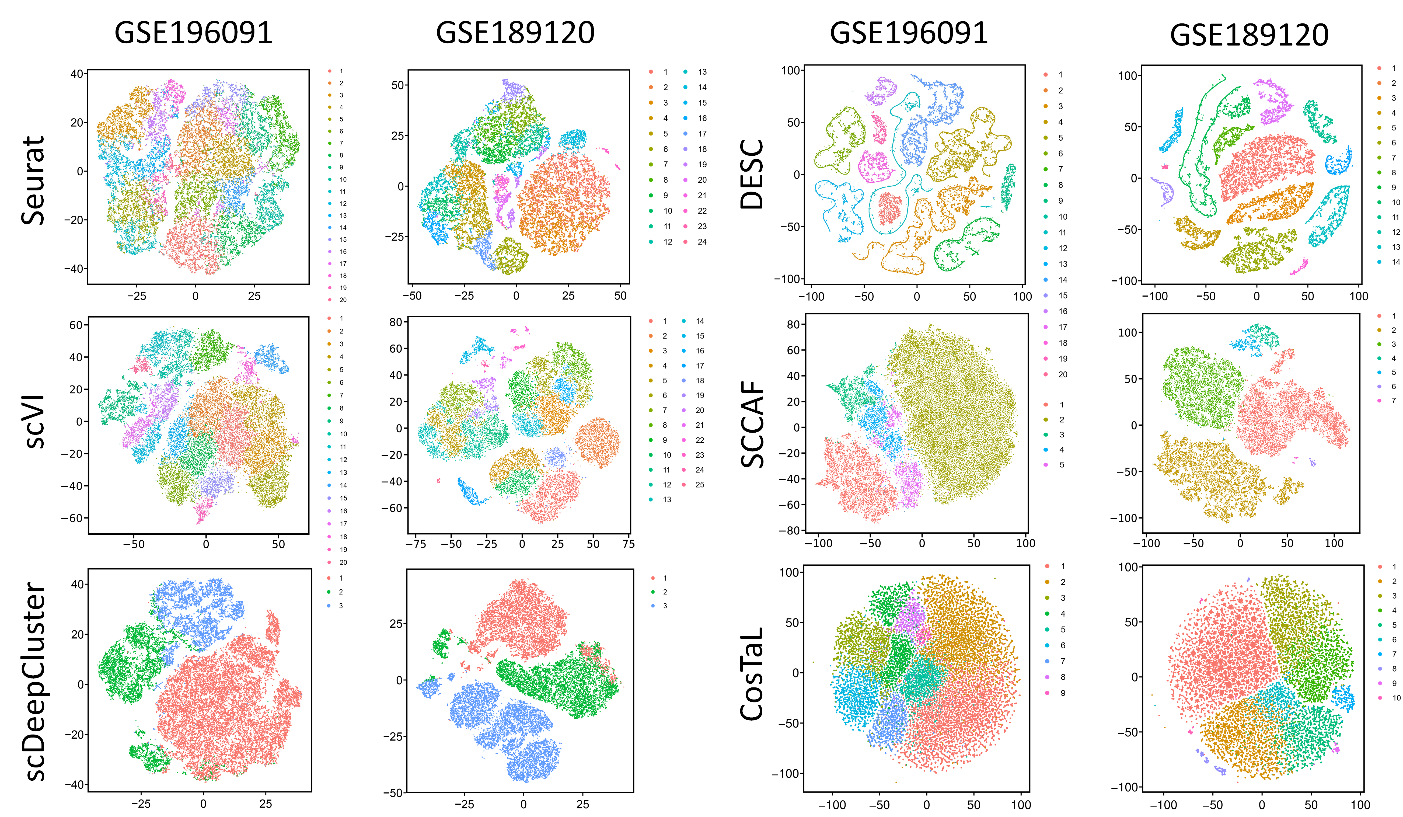


**Supplementary Figure S2**. The t-SNE plots generated using seven clustering algorithms for Type 2 data. Different colors represent clusters generated by clustering algorithms.

**
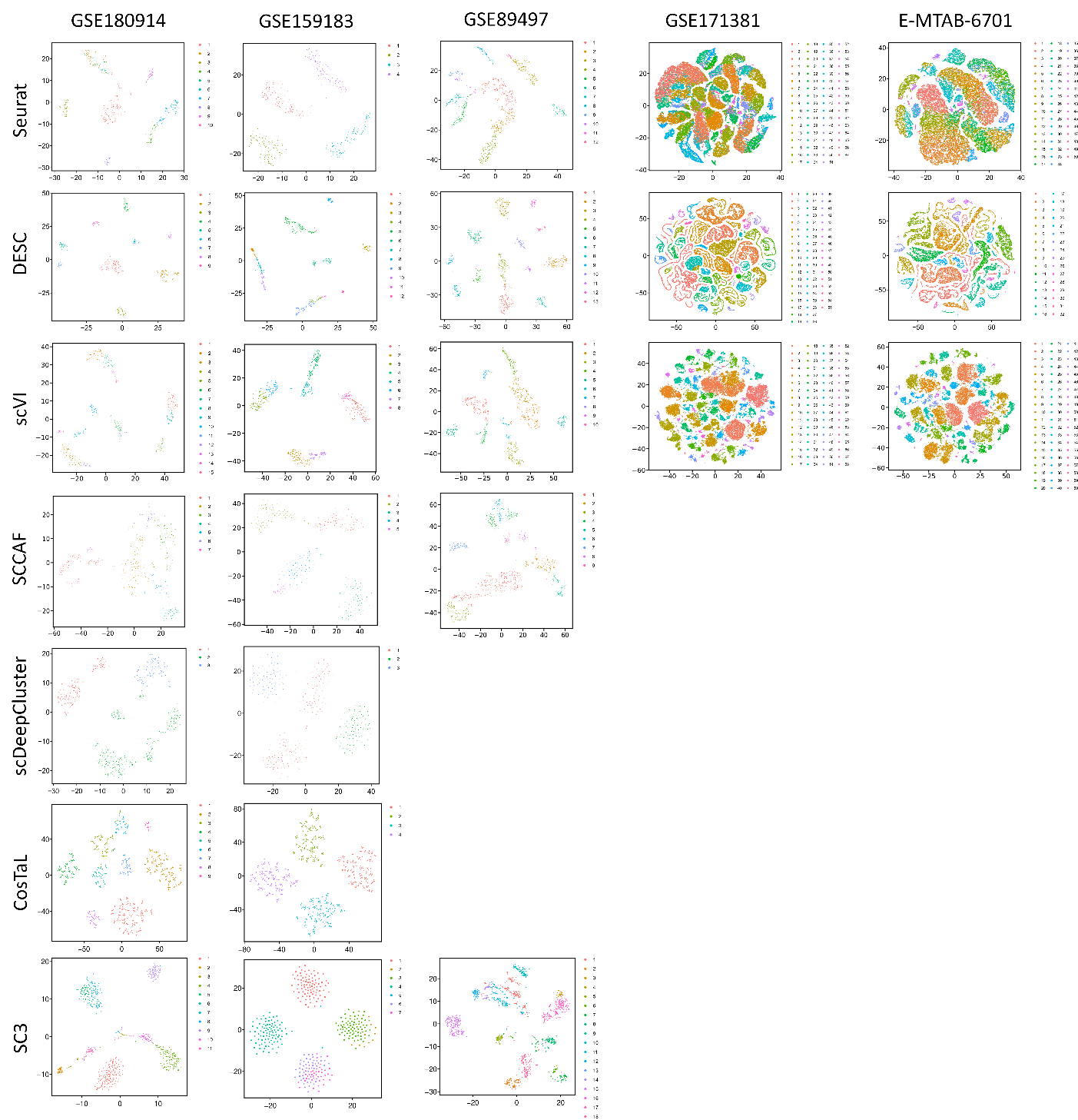
**

**Supplementary Figure S3**. The t-SNE plots generated using seven clustering algorithms for Type 3 data. Different colors represent clusters generated by clustering algorithms.

**
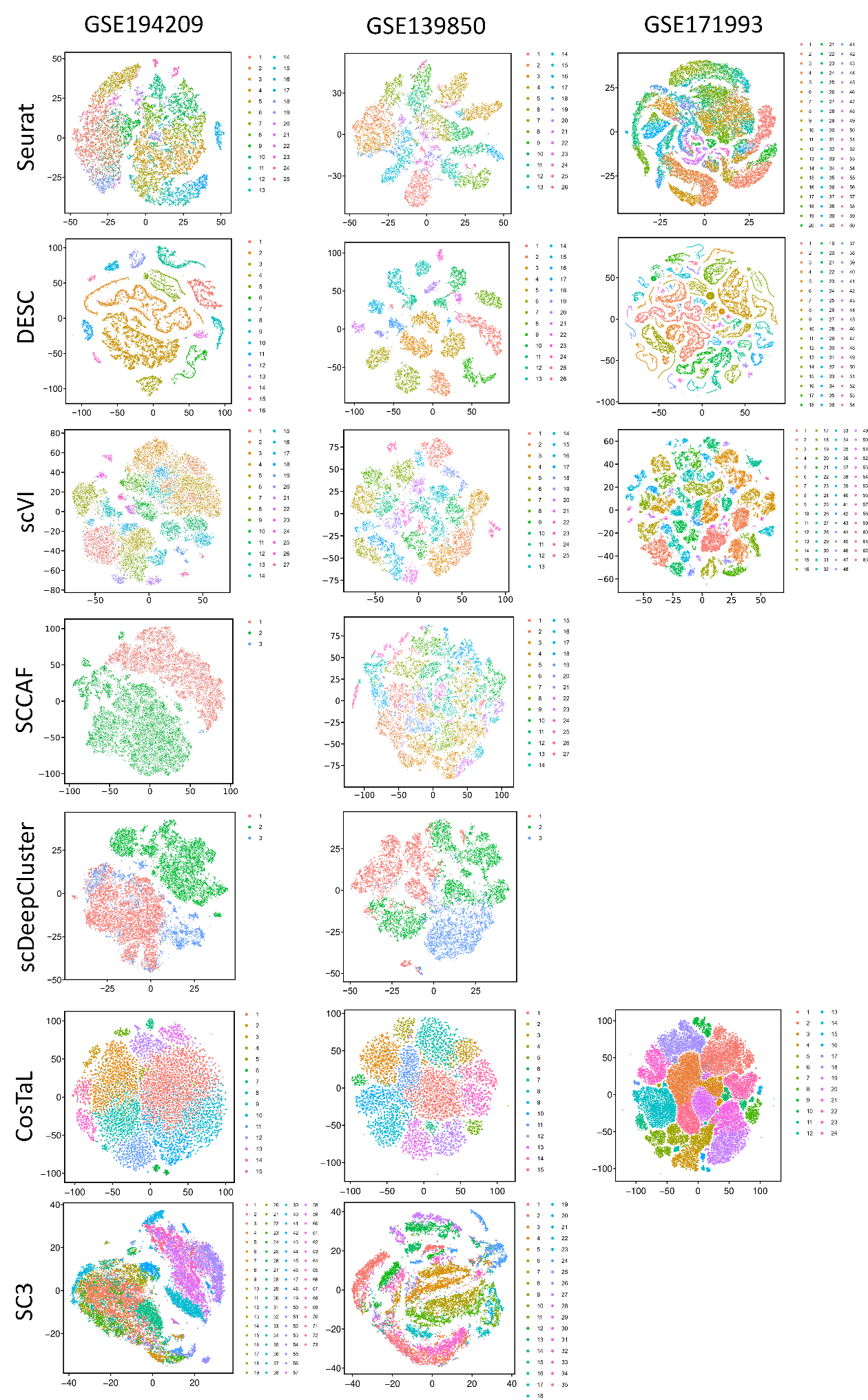
**

**Supplementary Table S1**. Seven clustering algorithms were utilized for comparative analysis. The core characteristics and advantages of each algorithm, as well as their limitations are described.

| **Algorithm**  **(Development**  **Environment)** | **Advantages & Characteristics** | **Limitations & Shortcomings** | |
| --- | --- | --- | --- |
| DESC  (Python) | DESC balances clustering accuracy and stability, occupies less memory, and does not require the removal of batch effects [14]. | | DESC rely on pure data-driven, which cannot ensure that the number of clusters identified is meaningful in biology [26]. |
| Seurat  (R) | Seurat has significantly improved the ability to define cell states across multiple biological contexts and data types [27]. | | Seurat may oversimplify the genetic relationship between complex cells and the overall cell population [26]. |
| scDeepCluster  (Python+R) | scDeepCluster enhances the capability to acquire robust feature representations of data by denoising autoencoder technology [6, 20]. | | scDeepCluster generates clustering results based on a pre-set number of clusters, causing significant deviations between cell clusters and real cell types [28]. |
| scVI  (Python) | scVI provides the possibility to use negative binomial distribution to learn scRNA-seq data and add zero inflation terms [15]. | | scVI cannot directly deal with Non-Euclidean spatial data like cell graphs [18]. |
| SCCAF  (Python) | SCCAF uses a "transparent box" classifier to accurately reconstruct cell annotations in different scRNA-seq datasets [22]. | | SCCAF performs less efficiently when dealing with continuous biological processes [29]. |
| **CosTaL**  (Python) | CosTaL streamlines scRNA-seq clustering by eliminating normalization, scaling, and PCA transformation, reducing parameter selection work and enhancing efficiency [25]. | | CosTaL exclusively supports cosine similarity and is not compatible with PCA transformations [25]. |
| **SC3**  (R) | SC3 utilizes a parallelisation approach, whereby a significant subset of the parameter space is evaluated simultaneously to obtain a set of clustering [23]. | | SC3 clustering algorithm may face the problems of computational complexity and memory consumption when dealing with large-scale data sets [24, 30]. |

**Supplementary Table S2**. Nine evaluation metrics were utilized for comparing performance of clustering algorithms.

| **Metrics** | **Descriptions** |
| --- | --- |
| **ARI** | ARI is commonly used to measure the degree of agreement between two data partitions in clustering analysis [31]. The ARI score ranges from -1 to 1, and the larger the value, the higher the consistency between the clustering results and the real labels [32]. |
| **H Score** | H Score is useful to measure how much the cells in a cluster are similar [33]. H Score is calculated based on conditional entropy and joint entropy [34]. The H Score ranges from 0 to 1, and the larger the value, the higher the consistency between the clustering results and the real labels [32]. |
| **KL** | The KL is a metric to measure the performance of clustering algorithm for batch effect removal [14], KL provides relative entropy, which can be used to measure the disorder of a data set which usually ranges between 0 and 1[35]. Smaller KL indicates the better performance in batch effect removal [14]. |
| **FMI** | FMI is an external evaluation metric to determine the similarity between two clusterings [36]. The FMI score of random clustering is 0, regardless of the number of clusters or individuals [32]. Its disadvantage is that you must know the true label before you can use it [32]. |
| **AMI** | AMI is closely related to variation of information [37]. Perfect labeling has an AMI of 1, and random labeling has an AMI close to 0. Values close to 0 indicate that the labels are largely independent, when values close to 1 indicate a significant agreement between the two clustering [32]. |
| **NMI** | NMI is an external evaluation metric to determine the similarity between two clusterings. NMI is a normalization of the Mutual Information. The value range of NMI score is between 0 and 1, and the larger the value, the higher the consistency between the clustering results and the real labels [32]. |
| **V-measure** | V-measure is an entropy-based metric that explicitly assesses the similarity between clustering results and real labels [33]. V-measure is calculated as the harmonic mean of distinct homogeneity and completeness scores. The V-measure score ranges from 0 to 1, and the larger the value, the higher the consistency between the clustering results and the real labels [32]. |
| **HF1 Score** | HF1 is F1 score-based metric that explicitly evaluate performance of clustering algorithm [25]. HF1 is calculated based on the Hungarian algorithm, which is a combinatorial optimization method that identifies the assignment with the lowest F-measure in true cell populations [38]. The HF1 score range from 0 to 1, with higher values indicating greater consistency between the clustering results and the real labels [25]. |
| **FF1 Score** | FF1 is F1 score-based metric that explicitly evaluate the performance of clustering algorithm [25]. FF1 is calculated based on the FlowCAPI methodology [39]. The FF1 score range from 0 to 1, with higher values indicating greater consistency between the clustering results and the real labels [25]. |

**Supplementary Table S3**. ARI values of seven clustering algorithms across ten scRNA-seq datasets. NA indicates that the ARI value of clustering algorithm is not available.

| **Dataset ID** | **Type** | **Seurat** | **DESC** | **scVI** | **scDeepcluster** | **SCCAF** | **CosTaL** | **SC3** |
| --- | --- | --- | --- | --- | --- | --- | --- | --- |
| **GSE196091** | Type1 | 0.38862 | 0.36317 | 0.40362 | 0.43413 | 0.16836 | 0.33958 | NA(memory) |
| **GSE189120** | Type1 | 0.96899 | 0.94306 | 0.93920 | 0.86177 | 0.80753 | 0.97448 | NA(memory) |
| **GSE180914** | Type2 | 0.86236 | 0.86520 | 0.87526 | 0.42784 | 0.63403 | 0.92257 | 0.86730 |
| **GSE159183** | Type2 | 0.99276 | 0.98917 | 0.98920 | 0.70265 | 0.91824 | 0.99276 | 0.97834 |
| **GSE89497** | Type2 | 0.59617 | 0.60841 | 0.53134 | NA(TPM) | 0.33135 | NA(TPM) | 0.59422 |
| **GSE171381** | Type2 | 0.60858 | 0.48294 | 0.59421 | NA(memory) | NA(memory) | NA(memory) | NA(memory) |
| **E-MTAB-6701** | Type2 | 0.68362 | 0.73538 | 0.19974 | NA(memory) | NA(memory) | NA(memory) | NA(memory) |
| **GSE194209** | Type3 | 0.00845 | 0.00254 | 0.01114 | 0.00080 | 0.00117 | 0.00294 | 0.01912 |
| **GSE139850** | Type3 | 0.81955 | 0.82310 | 0.78135 | 0.24974 | 0.20681 | 0.83749 | 0.78243 |
| **GSE171993** | Type3 | 0.82517 | 0.81595 | 0.22347 | NA(memory) | NA(memory) | 0.80124 | NA(memory) |

**Supplementary Table S4**. FMI of seven clustering algorithms across ten scRNA-seq datasets. NA indicates that the FMI value of clustering algorithm is not available.

| **Dataset ID** | **Type** | **Seurat** | **DESC** | **scVI** | **scDeepcluster** | **SCCAF** | **CosTaL** | **SC3** |
| --- | --- | --- | --- | --- | --- | --- | --- | --- |
| **GSE196091** | Type1 | 0.49721 | 0.56113 | 0.57370 | 0.67193 | 0.55927 | 0.52095 | NA(memory) |
| **GSE189120** | Type1 | 0.97944 | 0.96227 | 0.95970 | 0.90818 | 0.87249 | 0.98308 | NA(memory) |
| **GSE180914** | Type2 | 0.88700 | 0.88889 | 0.89749 | 0.60954 | 0.70969 | 0.93600 | 0.89176 |
| **GSE159183** | Type2 | 0.99456 | 0.99186 | 0.99189 | 0.80840 | 0.91677 | 0.99456 | 0.98372 |
| **GSE89497** | Type2 | 0.70474 | 0.71722 | 0.66115 | NA(TPM) | 0.54420 | NA(TPM) | 0.70464 |
| **GSE171381** | Type2 | 0.63962 | 0.53051 | 0.62755 | NA(memory) | NA(memory) | NA(memory) | NA(memory) |
| **E-MTAB-6701** | Type2 | 0.71811 | 0.77261 | 0.30065 | NA(memory) | NA(memory) | NA(memory) | NA(memory) |
| **GSE194209** | Type3 | 0.50464 | 0.57005 | 0.51687 | 0.50933 | 0.50729 | 0.54639 | 0.51220 |
| **GSE139850** | Type3 | 0.90781 | 0.90975 | 0.88911 | 0.66110 | 0.59724 | 0.91603 | 0.88918 |
| **GSE171993** | Type3 | 0.83758 | 0.82842 | 0.28184 | NA(memory) | NA(memory) | 0.82236 | NA(memory) |

**Supplementary Table S5**. HF1 Scores of seven clustering algorithms across ten scRNA-seq datasets. NA indicates that the HF1 Scores value of clustering algorithm is not available.

| **Dataset ID** | **Type** | **Seurat** | **DESC** | **scVI** | **scDeepcluster** | **SCCAF** | **CosTaL** | **SC3** |
| --- | --- | --- | --- | --- | --- | --- | --- | --- |
| **GSE196091** | Type1 | 0.60767 | 0.45534 | 0.69809 | 0.35791 | 0.30056 | 0.61696 | NA(memory) |
| **GSE189120** | Type1 | 0.98942 | 0.97896 | 0.97748 | 0.81770 | 0.73611 | 0.99142 | NA(memory) |
| **GSE180914** | Type2 | 0.91651 | 0.89268 | 0.89581 | 0.31937 | 0.68064 | 0.94963 | 0.90957 |
| **GSE159183** | Type2 | 0.79784 | 0.79674 | 0.79675 | 0.53068 | 0.78620 | 0.79783 | 0.79350 |
| **GSE89497** | Type2 | 0.82579 | 0.83650 | 0.81036 | NA(TPM) | 0.53740 | NA(TPM) | 0.83983 |
| **GSE171381** | Type2 | 0.66055 | 0.47485 | 0.67286 | NA(memory) | NA(memory) | NA(memory) | NA(memory) |
| **E-MTAB-6701** | Type2 | 0.59561 | 0.58953 | 0.10711 | NA(memory) | NA(memory) | NA(memory) | NA(memory) |
| **GSE194209** | Type3 | 0.54590 | 0.48788 | 0.55665 | 0.51025 | 0.51426 | 0.50413 | 0.56800 |
| **GSE139850** | Type3 | 0.63417 | 0.63639 | 0.62780 | 0.48637 | 0.59331 | 0.69991 | 0.62793 |
| **GSE171993** | Type3 | 0.66286 | 0.62225 | 0.10169 | NA(memory) | NA(memory) | 0.55831 | NA(memory) |

**Supplementary Table S6**. FF1 Scores of seven clustering algorithms across ten scRNA-seq datasets. NA indicates that the FF1 Scores value of clustering algorithm is not available.

| **Dataset ID** | **Type** | **Seurat** | **DESC** | **scVI** | **scDeepcluster** | **SCCAF** | **CosTaL** | **SC3** |
| --- | --- | --- | --- | --- | --- | --- | --- | --- |
| **GSE196091** | Type1 | 0.62882 | 0.58127 | 0.66136 | 0.65900 | 0.57960 | 0.59684 | NA(memory) |
| **GSE189120** | Type1 | 0.98953 | 0.97977 | 0.97837 | 0.94865 | 0.93112 | 0.99145 | NA(memory) |
| **GSE180914** | Type2 | 0.91917 | 0.91007 | 0.91318 | 0.62205 | 0.77069 | 0.95276 | 0.91689 |
| **GSE159183** | Type2 | 0.99464 | 0.99329 | 0.99329 | 0.82722 | 0.95865 | 0.99464 | 0.98925 |
| **GSE89497** | Type2 | 0.82611 | 0.82955 | 0.79772 | NA(TPM) | 0.65431 | NA(TPM) | 0.82825 |
| **GSE171381** | Type2 | 0.74232 | 0.62064 | 0.72601 | NA(memory) | NA(memory) | NA(memory) | NA(memory) |
| **E-MTAB-6701** | Type2 | 0.73847 | 0.79494 | 0.34676 | NA(memory) | NA(memory) | NA(memory) | NA(memory) |
| **GSE194209** | Type3 | 0.54592 | 0.60674 | 0.55673 | 0.54339 | 0.53803 | 0.50413 | 0.56804 |
| **GSE139850** | Type3 | 0.92565 | 0.92651 | 0.91583 | 0.71298 | 0.70397 | 0.93361 | 0.91601 |
| **GSE171993** | Type3 | 0.82801 | 0.83196 | 0.31038 | NA(memory) | NA(memory) | 0.80232 | NA(memory) |

**Supplementary Table S7**. H Score of seven clustering algorithms across ten scRNA-seq datasets. NA indicates that the H Score value of clustering algorithm is not available.

| **Dataset ID** | **Type** | **Seurat** | **DESC** | **scVI** | **scDeepcluster** | **SCCAF** | **CosTaL** | **SC3** |
| --- | --- | --- | --- | --- | --- | --- | --- | --- |
| **GSE196091** | Type1 | 0.48122 | 0.43837 | 0.52194 | 0.39786 | 0.20316 | 0.47848 | NA(memory) |
| **GSE189120** | Type1 | 0.94413 | 0.91001 | 0.90081 | 0.81837 | 0.73487 | 0.95274 | NA(memory) |
| **GSE180914** | Type2 | 0.86236 | 0.86520 | 0.87526 | 0.46779 | 0.63463 | 0.92294 | 0.87609 |
| **GSE159183** | Type2 | 0.98330 | 0.97862 | 0.97864 | 0.73063 | 0.95695 | 0.98330 | 0.96375 |
| **GSE89497** | Type2 | 0.59617 | 0.60841 | 0.53134 | NA(TPM) | 0.38356 | NA(TPM) | 0.63357 |
| **GSE171381** | Type2 | 0.68896 | 0.56960 | 0.69150 | NA(memory) | NA(memory) | NA(memory) | NA(memory) |
| **E-MTAB-6701** | Type2 | 0.75995 | 0.78929 | 0.21203 | NA(memory) | NA(memory) | NA(memory) | NA(memory) |
| **GSE194209** | Type3 | 0.00614 | 0.00274 | 0.01485 | 0.00065 | 0.00092 | 0.00273 | 0.01398 |
| **GSE139850** | Type3 | 0.659 | 0.6657 | 0.63698 | 0.23737 | 0.15449 | 0.69377 | 0.62365 |
| **GSE171993** | Type3 | 0.82828 | 0.82707 | 0.15385 | NA(memory) | NA(memory) | 0.80622 | NA(memory) |

**Supplementary Table S8**. AMI of seven clustering algorithms across ten scRNA-seq datasets. NA indicates that the AMI value of clustering algorithm is not available.

| **Dataset ID** | **Type** | **Seurat** | **DESC** | **scVI** | **scDeepcluster** | **SCCAF** | **CosTaL** | **SC3** |
| --- | --- | --- | --- | --- | --- | --- | --- | --- |
| **GSE196091** | Type1 | 0.49716 | 0.47882 | 0.52356 | 0.52667 | 0.30052 | 0.47172 | NA(memory) |
| **GSE189120** | Type1 | 0.94479 | 0.91114 | 0.90166 | 0.81769 | 0.73609 | 0.95330 | NA(memory) |
| **GSE180914** | Type2 | 0.87416 | 0.85286 | 0.87221 | 0.59780 | 0.66870 | 0.92286 | 0.88636 |
| **GSE159183** | Type2 | 0.98851 | 0.98379 | 0.98380 | 0.83842 | 0.91615 | 0.98851 | 0.96877 |
| **GSE89497** | Type2 | 0.62106 | 0.63345 | 0.55855 | NA(TPM) | 0.41714 | NA(TPM) | 0.62985 |
| **GSE171381** | Type2 | 0.70327 | 0.59955 | 0.70584 | NA(memory) | NA(memory) | NA(memory) | NA(memory) |
| **E-MTAB-6701** | Type2 | 0.79537 | 0.84439 | 0.25014 | NA(memory) | NA(memory) | NA(memory) | NA(memory) |
| **GSE194209** | Type3 | 0.00612 | 0.00305 | 0.01127 | 0.00063 | 0.00090 | 0.00290 | 0.01400 |
| **GSE139850** | Type3 | 0.70891 | 0.71631 | 0.68845 | 0.28006 | 0.16176 | 0.72087 | 0.67238 |
| **GSE171993** | Type3 | 0.84123 | 0.84304 | 0.28192 | NA(memory) | NA(memory) | 0.85689 | NA(memory) |

**Supplementary Table S9**. NMI of seven clustering algorithms across ten scRNA-seq datasets. NA indicates that the NMI value of clustering algorithm is not available.

| **Dataset ID** | **Type** | **Seurat** | **DESC** | **scVI** | **scDeepcluster** | **SCCAF** | **CosTaL** | **SC3** |
| --- | --- | --- | --- | --- | --- | --- | --- | --- |
| **GSE196091** | Type1 | 0.49721 | 0.47886 | 0.52362 | 0.52669 | 0.30056 | 0.47178 | NA(memory) |
| **GSE189120** | Type1 | 0.94479 | 0.91115 | 0.90167 | 0.81770 | 0.73611 | 0.95330 | NA(memory) |
| **GSE180914** | Type2 | 0.87605 | 0.85506 | 0.87413 | 0.60031 | 0.67305 | 0.92401 | 0.88808 |
| **GSE159183** | Type2 | 0.98858 | 0.98389 | 0.98390 | 0.83918 | 0.91677 | 0.98858 | 0.96896 |
| **GSE89497** | Type2 | 0.62244 | 0.63481 | 0.56019 | NA(TPM) | 0.41889 | NA(TPM) | 0.63121 |
| **GSE171381** | Type2 | 0.70350 | 0.59985 | 0.70608 | NA(memory) | NA(memory) | NA(memory) | NA(memory) |
| **E-MTAB-6701** | Type2 | 0.79581 | 0.84469 | 0.25074 | NA(memory) | NA(memory) | NA(memory) | NA(memory) |
| **GSE194209** | Type3 | 0.00615 | 0.00309 | 0.01131 | 0.00066 | 0.00093 | 0.00293 | 0.01404 |
| **GSE139850** | Type3 | 0.70893 | 0.71635 | 0.68847 | 0.28013 | 0.16191 | 0.72092 | 0.67241 |
| **GSE171993** | Type3 | 0.84163 | 0.84340 | 0.28272 | NA(memory) | NA(memory) | 0.85718 | NA(memory) |

**Supplementary Table S10**. V-measure of seven clustering algorithms across ten scRNA-seq datasets. NA indicates that the V-measure value of clustering algorithm is not available.

| **Dataset ID** | **Type** | **Seurat** | **DESC** | **scVI** | **scDeepcluster** | **SCCAF** | **CosTaL** | **SC3** |
| --- | --- | --- | --- | --- | --- | --- | --- | --- |
| **GSE196091** | Type1 | 0.49721 | 0.47886 | 0.52362 | 0.52669 | 0.30056 | 0.47178 | NA(memory) |
| **GSE189120** | Type1 | 0.94479 | 0.91115 | 0.90167 | 0.81770 | 0.73611 | 0.95330 | NA(memory) |
| **GSE180914** | Type2 | 0.87605 | 0.85506 | 0.87413 | 0.60031 | 0.67305 | 0.92401 | 0.88808 |
| **GSE159183** | Type2 | 0.98858 | 0.98389 | 0.98390 | 0.83918 | 0.91677 | 0.98858 | 0.96896 |
| **GSE89497** | Type2 | 0.62244 | 0.63481 | 0.56019 | NA(TPM) | 0.41889 | NA(TPM) | 0.63121 |
| **GSE171381** | Type2 | 0.70350 | 0.59985 | 0.70608 | NA(memory) | NA(memory) | NA(memory) | NA(memory) |
| **E-MTAB-6701** | Type2 | 0.79581 | 0.84469 | 0.25074 | NA(memory) | NA(memory) | NA(memory) | NA(memory) |
| **GSE194209** | Type3 | 0.00615 | 0.00309 | 0.01131 | 0.00066 | 0.00093 | 0.00293 | 0.01404 |
| **GSE139850** | Type3 | 0.70893 | 0.71635 | 0.68847 | 0.28013 | 0.16191 | 0.72092 | 0.67241 |
| **GSE171993** | Type3 | 0.84163 | 0.84340 | 0.28272 | NA(memory) | NA(memory) | 0.85718 | NA(memory) |

**Supplementary Table S11**. KL of seven clustering algorithms across ten scRNA-seq datasets. NA indicates that the KL value of clustering algorithm is not available.

| **Dataset ID** | **Type** | **Seurat** | **DESC** | **scVI** | **scDeepcluster** | **SCCAF** | **CosTaL** | **SC3** |
| --- | --- | --- | --- | --- | --- | --- | --- | --- |
| **GSE196091** | Type1 | 0.16099 | 0.18338 | 0.16402 | 0.12683 | 0.47660 | 0.18267 | NA(memory) |
| **GSE189120** | Type1 | 0.00236 | 0.00371 | 0.00457 | 0.01063 | 0.01523 | 0.00199 | NA(memory) |
| **GSE180914** | Type2 | 0.00872 | 0.03089 | 0.03170 | 0.36189 | 0.16713 | 0.00312 | 0.00579 |
| **GSE159183** | Type2 | 0.00095 | 0.00250 | 0.00191 | 0.09833 | 0.29728 | 0.00034 | 0.00468 |
| **GSE89497** | Type2 | 0.04497 | 0.04392 | 0.05938 | NA(TPM) | 0.08613 | NA(TPM) | 0.04622 |
| **GSE171381** | Type2 | 0.11319 | 0.19492 | 0.14226 | NA(memory) | NA(memory) | NA(memory) | NA(memory) |
| **E-MTAB-6701** | Type2 | 0.01788 | 0.04693 | 0.17804 | NA(memory) | NA(memory) | NA(memory) | NA(memory) |
| **GSE194209** | Type3 | 0.10418 | 0.09802 | 0.09675 | 0.10771 | 0.11202 | 0.10323 | 0.09755 |
| **GSE139850** | Type3 | 0.02365 | 0.02244 | 0.01927 | 0.04819 | 0.06533 | 0.02153 | 0.01970 |
| **GSE171993** | Type3 | 0.03730 | 0.03260 | 0.18679 | NA(memory) | NA(memory) | 0.12060 | NA(memory) |

**Supplementary Table S12**. Computation speed and memory usage were assessed across seven clustering algorithms using ten scRNA-seq datasets. All performance tests are run on a machine with 251G RAM and 2.00GHz CPUs.

| **Dataset ID** | **Seurat** | **DESC** | **scDeepCluster** | **scVI** | **SCCAF** | **CosTaL** | **SC3** |
| --- | --- | --- | --- | --- | --- | --- | --- |
| **GSE180914**  (681) | CS: <0.1min | CS: 0.4min | CS: 6min | CS: 3min | CS: 2min | CS: >0.1min | CS: 3min |
|  | MEM: 356Mb | MEM: 472Mb | MEM: 737Mb | MEM: 527Mb | MEM: 375Mb | MEM: 598MB | MEM: 2943Mb |
| **GSE159183**  (738) | CS: <0.1min | CS: 0.1min | CS: 10min | CS: 6min | CS: 3min | CS: >0.1min | CS: 2min |
|  | MEM: 444Mb | MEM: 412Mb | MEM: 1,007Mb | MEM: 577Mb | MEM: 481Mb | MEM: 669Mb | MEM: 3,399Mb |
| **GSE89497**  (1567) | CS: <0.1min | CS: 0.1min | CS: NA | CS: 11min | CS: 7min | CS: NA | CS: 2min |
|  | MEM: 627Mb | MEM: 595Mb | MEM: NA | MEM: 780Mb | MEM: 562Mb | MEM: NA | MEM: 3,942Mb |
| **GSE139850**  (14874) | CS: <0.1min | CS: 3min | CS: 183min | CS: 101min | CS: 14min | CS: 5min | CS: 126min |
|  | MEM: 1,651Mb | MEM: 1,329Mb | MEM: 2,944Mb | MEM: 1,682Mb | MEM: 1,419Mb | MEM: 733 Mb | MEM: >10GB |
| **GSE194209**  (20393) | CS: 0.5min | CS: 6min | CS: 196min | CS: 193min | CS: 20min | CS: 36min | CS: >720min |
|  | MEM: 1,582Mb | MEM: 1,348Mb | MEM: 3,115Mb | MEM: 3,036Mb | MEM: 1,236Mb | MEM: 904Mb | MEM: >10GB |
| **GSE189120**  (25511) | CS: 0.5min | CS: 5min | CS: 293min | CS: 164min | CS: 20min | CS: 50min | CS: NA |
|  | MEM: 2,511Mb | MEM: 1,772Mb | MEM: 4,428Mb | MEM: 2,400Mb | MEM: 2000Mb | MEM: 1,128Mb | MEM: NA |
| **GSE196091**  (31730) | CS: 1min | CS: 6min | CS: >300min | CS: 193min | CS: 25min | CS: 66min | CS: NA |
|  | MEM: 3,406Mb | MEM: 1,612Mb | MEM: >4GB | MEM: 2755Mb | MEM: 2,057Mb | MEM: 1500Mb | MEM: NA |
| **GSE171993**  (52834) | CS: 2min | CS: 18min | CS: >300min | CS: >200min | CS: NA | CS: 197min | CS: NA |
|  | MEM: 2,843Mb | MEM: 2,575Mb | MEM: >4GB | MEM: >3GB | MEM: NA | MEM: >3Gb | MEM: NA |
| **E-MTAB-6701**  (64734) | CS: 2min | CS: 17min | CS: NA | CS: >200min | CS: NA | CS: NA | CS: NA |
|  | MEM: 4,952Mb | MEM: 3,639Mb | MEM: NA | MEM: >3GB | MEM: NA | MEM: NA | MEM: NA |
| **GSE171381**  (83378) | CS: 3min | CS: 13min | CS: NA | CS: >200min | CS: NA | CS: NA | CS: NA |
|  | MEM: 4,770 Mb | MEM: 2043 Mb | MEM: NA | MEM: >3GB | MEM: NA | MEM: NA | MEM: NA |

**References**

1. Falcinelli SD, Peterson JJ, Turner AW et al. Combined noncanonical NF-kappaB agonism and targeted BET bromodomain inhibition reverse HIV latency ex vivo. J Clin Invest 2022;132.

2. Ghanem MH, Shih AJ, Khalili H et al. Proteomic and Single-Cell Transcriptomic Dissection of Human Plasmacytoid Dendritic Cell Response to Influenza Virus. Front Immunol 2022;13:814627.

3. Chen S, Gao Y, Fan Y et al. The Dynamic Change of Immune Responses Between Acute and Recurrence Stages of Rodent Malaria Infection. Front Microbiol 2022;13:844975.

4. Satija R, Farrell JA, Gennert D et al. Spatial reconstruction of single-cell gene expression data. Nat Biotechnol 2015;33:495-502.

5. Peng YR, Shekhar K, Yan W et al. Molecular Classification and Comparative Taxonomics of Foveal and Peripheral Cells in Primate Retina. Cell 2019;176:1222-1237 e1222.

6. Tian T, Wan J, Song Q et al. Clustering single-cell RNA-seq data with a model-based deep learning approach. Nature Machine Intelligence 2019;1:191-198.

7. Vento-Tormo R, Efremova M, Botting RA et al. Single-cell reconstruction of the early maternal-fetal interface in humans. Nature 2018;563:347-353.

8. Chen Y, Huang H, Li Y et al. TIGIT Blockade Exerts Synergistic Effects on Microwave Ablation Against Cancer. Front Immunol 2022;13:832230.

9. Philip Creamer J, Luff SA, Yu H et al. CD1d expression demarcates CDX4+ hemogenic mesoderm with definitive hematopoietic potential. Stem Cell Res 2022;62:102808.

10. Liang Y, Kaneko K, Xin B et al. Temporal analyses of postnatal liver development and maturation by single-cell transcriptomics. Dev Cell 2022;57:398-414 e395.

11. Collin J, Queen R, Zerti D et al. A single cell atlas of human cornea that defines its development, limbal progenitor cells and their interactions with the immune cells. Ocul Surf 2021;21:279-298.

12. Papazoglou A, Huang M, Bulik M et al. Epigenetic Regulation of Profibrotic Macrophages in Systemic Sclerosis-Associated Interstitial Lung Disease. Arthritis Rheumatol 2022;74:2003-2014.

13. Ionkina AA, Balderrama-Gutierrez G, Ibanez KJ et al. Transcriptome analysis of heterogeneity in mouse model of metastatic breast cancer. Breast Cancer Res 2021;23:93.

14. Li X, Wang K, Lyu Y et al. Deep learning enables accurate clustering with batch effect removal in single-cell RNA-seq analysis. Nat Commun 2020;11:2338.

15. Lopez R, Regier J, Cole MB et al. Deep generative modeling for single-cell transcriptomics. Nat Methods 2018;15:1053-1058.

16. Bollback JP. Bayesian model adequacy and choice in phylogenetics. Mol Biol Evol 2002;19:1171-1180.

17. Medvedovic M, Yeung KY, Bumgarner RE. Bayesian mixture model based clustering of replicated microarray data. Bioinformatics 2004;20:1222-1232.

18. Xu C, Cai L, Gao J. An efficient scRNA-seq dropout imputation method using graph attention network. BMC Bioinformatics 2021;22:582.

19. Luecken MD, Buttner M, Chaichoompu K et al. Benchmarking atlas-level data integration in single-cell genomics. Nat Methods 2022;19:41-50.

20. Vincent P, Larochelle H, Lajoie I et al. Stacked Denoising Autoencoders: Learning Useful Representations in a Deep Network with a Local Denoising Criterion. Journal of Machine Learning Research 2010;11:3371-3408.

21. Eraslan G, Simon LM, Mircea M et al. Single-cell RNA-seq denoising using a deep count autoencoder. Nat Commun 2019;10:390.

22. Miao Z, Moreno P, Huang N et al. Putative cell type discovery from single-cell gene expression data. Nat Methods 2020;17:621-628.

23. Kiselev VY, Kirschner K, Schaub MT et al. SC3: consensus clustering of single-cell RNA-seq data. Nat Methods 2017;14:483-486.

24. Kiselev VY, Andrews TS, Hemberg M. Challenges in unsupervised clustering of single-cell RNA-seq data. Nat Rev Genet 2019;20:273-282.

25. Li Y, Nguyen J, Anastasiu DC et al. CosTaL: an accurate and scalable graph-based clustering algorithm for high-dimensional single-cell data analysis. Brief Bioinform 2023;24.

26. Zhao JP, Hou TS, Su Y et al. scSSA: A clustering method for single cell RNA-seq data based on semi-supervised autoencoder. Methods 2022;208:66-74.

27. Hao YH, Hao S, Andersen-Nissen E et al. Integrated analysis of multimodal single-cell data. Cell 2021;184:3573-+.

28. Wang H, Zhao J, Zheng C et al. scDSSC: Deep Sparse Subspace Clustering for scRNA-seq Data. PLoS Comput Biol 2022;18:e1010772.

29. Ren J, Zhang Q, Zhou Y et al. A downsampling method enables robust clustering and integration of single-cell transcriptome data. J Biomed Inform 2022;130:104093.

30. Tian T, Zhang J, Lin X et al. Model-based deep embedding for constrained clustering analysis of single cell RNA-seq data. Nat Commun 2021;12:1873.

31. Chacón JE, Rastrojo AI. Minimum adjusted Rand index for two clusterings of a given size. Advances in Data Analysis and Classification 2023;17:125-133.

32. Guyeux C, Chrétien S, Bou Tayeh G, et al. Introducing and Comparing Recent Clustering Methods for Massive Data Management in the Internet of Things. J Sens Actuator Netw 2019;8:56.

33. Hirschberg JB; Rosenberg A. V-Measure: A conditional entropy-based external cluster evaluation measure. Columbia j 2007;410-420.

34. Zhen CW, Wang YX, Geng JQ et al. A review and performance evaluation of clustering frameworks for single-cell Hi-C data. Brief Bioinform 2022;23.

35. Varma PS, Anand V. Fault-Tolerant indoor localization based on speed conscious recurrent neural network using Kullback-Leibler divergence. Peer Peer Netw Appl 2022;15:1370-1384.

36. Fowlkes, EB, Mallows, CL. A Method for Comparing Two Hierarchical Clusterings. J Am Stat Assoc 1983;78:553-569.

37. Meila M. Comparing clusterings - an information based distance. Journal of Multivariate Analysis 2007;98:873-895.

38. Samusik N, Good Z, Spitzer MH et al. Automated mapping of phenotype space with single-cell data. Nat Methods 2016;13:493-496.

39. Weber LM, Robinson MD. Comparison of clustering methods for high-dimensional single-cell flow and mass cytometry data. Cytometry A 2016;89:1084-1096.
